# Similarities in biological processes can be used to bridge ecology and molecular biology

**DOI:** 10.1111/eva.12961

**Published:** 2020-04-13

**Authors:** Johan Hallin, Angel F. Cisneros, Mathieu Hénault, Anna Fijarczyk, Rohan Dandage, Carla Bautista, Christian R. Landry

**Affiliations:** ^1^ Département de biochimie de microbiologie et de bio‐informatique Faculté des sciences et de génie Université Laval Québec Canada; ^2^ Département de biologie Faculté des sciences et de génie Université Laval Québec Canada; ^3^ Institut de Biologie Intégrative et des Systèmes (IBIS) Université Laval Québec Canada; ^4^ PROTEO Le réseau québécois de recherche sur la fonction la structure et l’ingénierie des protéines Université Laval Québec Canada; ^5^ Centre de Recherche en Données Massives (CRDM) Université Laval Québec Canada

**Keywords:** ecology, evolution, gene duplication, molecular biology, networks, parasites, transposable elements

## Abstract

Much of the research in biology aims to understand the origin of diversity. Naturally, ecological diversity was the first object of study, but we now have the necessary tools to probe diversity at molecular scales. The inherent differences in how we study diversity at different scales caused the disciplines of biology to be organized around these levels, from molecular biology to ecology. Here, we illustrate that there are key properties of each scale that emerge from the interactions of simpler components and that these properties are often shared across different levels of organization. This means that ideas from one level of organization can be an inspiration for novel hypotheses to study phenomena at another level. We illustrate this concept with examples of events at the molecular level that have analogs at the organismal or ecological level and vice versa. Through these examples, we illustrate that biological processes at different organization levels are governed by general rules. The study of the same phenomena at different scales could enrich our work through a multidisciplinary approach, which should be a staple in the training of future scientists.

Louis Bernatchez gave Christian his first glimpse at how the sequence of genes can tell the history of a population or a species in a class on Molecular Ecology at Université Laval. After his undergraduate studies, Christian was interested in studying human population genetics and Louis gave him the contact information of some laboratories where he could join a graduate program to pursue this. However, all the laboratories he contacted advised him to first do a MSc with Louis before starting his graduate studies; advice that in hindsight was indeed very good. Between 1998 and 2000, Christian did his master's research in Louis’ laboratory, investigating the diversity of genes in the Major Histocompatibility Complex. This was Louis’ first project looking at nuclear coding gene sequences, most other projects were using “neutral” markers (e.g., microsatellites) to make inferences about population structure. Christian's main interest in biology was the study of evolution, and his work under the guidance of Louis’ was enlightening as this is where he discovered that evolution could be studied directly, not only inferred. After completing his MSc program and spending a few months as a research professional in Louis’ laboratory, Christian went on to enter a PhD program at Harvard University (not in human population genetics, by the way). Now as an independent PI of just over ten years, the early guidance of Louis is still an inspiration in how to create and sustain a healthy and productive research team, without sacrificing a well‐rounded and happy personal life.

## 1 INTRODUCTION

We work on several aspects of evolution using a range of methods. Among other things, we are interested in how genes are born (Durand et al., 2019; Nielly‐Thibault & Landry, 2019), how within‐genome dynamics affect evolution (Eberlein et al., 2019; Leducq et al., 2016), how genomes are molded in parasitic relationships (Hébert, Grambauer, Barber, Landry, & Aubin‐Horth, 2017), and how simple parts combine to form complex molecular systems (Diss, Ascencio, DeLuna, & Landry, 2014; Landry, Levy, Abd Rabbo, Tarassov, & Michnick, 2013). Because our team consists of people trained in different disciplines, from biophysics to biochemistry, microbiology, and ecology, we like to consider problems that span several levels of organization in terms of size, time, and space. Interestingly, the integration of these disciplines has helped us realize that living systems often show similar patterns regardless of the organization level, from the molecular to the organismal to the ecological. Using some examples from our own fields, we aim to exemplify how researchers in different fields and subfields can borrow concepts from each other to better understand and describe their own subject, and that these fields are not so different after all.

When a concept emerges in biology, or any other field, researchers may struggle to describe it using the language of that field. Since it is a new concept, the language of the field may fail to fully describe it or make it easily understandable. The description and understanding of a new concept can therefore be aided by borrowing language from other fields, where a similar concept exists and has been described. This was the case for Jacques Monod and François Jacob in 1961 when they introduced the term “program” to explain the lingering problem of how one single cell can develop into a complex organism through a seemingly static genome (Jacob & Monod, 1961). They borrowed the term program from the booming field of computational science, which had used it as a description of a sequence of commands given to a computer (Jacob, 1993; Keller, 2002). This was a computer science concept, turned genetics metaphor, turned concept again as the study of genetics went on, and it showcases the power of cross‐disciplinary knowledge to describe and to understand biological phenomena. However, carrying over metaphors from different fields could also oversimplify complex ideas, mask important details, and be confused with true descriptions; they should therefore be used consciously with these limitations in mind, and we should perhaps be open to updating our metaphors with time (Avise, 2001; Olson, Arroyo‐Santos, & Vergara‐Silva, 2019). We keep these limitations in mind when discussing the analogies we propose.

Within biology, comparing the different levels of organization can reveal common principles. In particular, some models applied to the macroscopic world can be applied to analogous microscopic phenomena (Francino, 2005). We can then use knowledge generated at one level to develop hypotheses that will inspire research at other levels. By doing so, we also argue that separating life‐science disciplines by the scale of the phenomena that are studied (as is done in many research institutions including our own, where microbiology and biology are taught in different programs and departments) might no longer be justified. In fact, new disciplines emerge by combining knowledge from different fields, which once happened with the advent of biochemistry and more recently with bioinformatics or synthetic biology. Since this allows for a more complete view of a given phenomenon, the training of future scientists should convey the importance of communication and exchange between fields.

In this article, we want to emphasize the importance of integrative science. We discuss how there are recurrent themes at the molecular and organismal levels, from how simple properties can give birth to systems with complex properties, to how new genes are born and how conflicts can arise between species and within genomes. We have written it with senior undergraduate students in mind and hope to inspire aspiring scientists (and established ones) to pursue exciting work in multidisciplinary and integrative research.

## 2 EMERGING PROPERTIES: HOW DO COMPLEX SYSTEMS EMERGE FROM SIMPLE PARTS?

**FIGURE 1:**
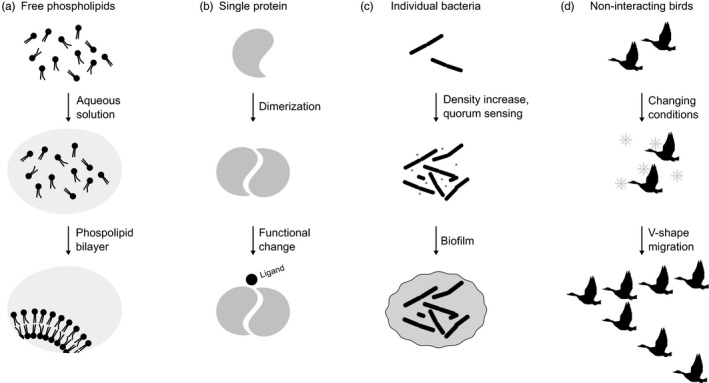
Emerging properties in systems with increasing complexity. (a) Free phospholipids self‐organize in aqueous solutions to form a bilayer. The pairwise rules are that the hydrophilic regions are attracted to the solvent, while the hydrophobic regions are repelled by it. This allows an efficient structure with a new functionality by encapsulating different materials, for instance, those of a cell. (b) The activity of some enzymes depends on their self‐assembly as dimers because their ligand binding site is located at the interface between the two copies. (c) Quorum sensing is a system that is activated when a certain threshold of cell density is achieved. Each cell reacts to each other, and this sum of interactions produces what is known as a biofilm, a new colonial form. An emergent property that arises in the colony can be, for example, a greater tolerance to antibiotics. (d) The V‐shaped migratory flight formation enables aerodynamic interactions between individuals so that they can conserve energy on long migration routes. This confers superior flight ability to the entire group compared to single individuals

Biologists often study the collective behavior of organisms, cells, or molecules. These collective behaviors are fascinating because they display properties that are not observable from the study of a single organism, cell, or molecule. Such emerging properties refer to new functionalities that arise from interactions between the elements of a system that would not be found in any of the parts acting in isolation (Johnson, 2006). Properties can emerge in an initially disordered system when interactions between individual elements promote organized behavior. To illustrate this, one can think of phospholipids. These amphipathic molecules self‐organize to form a bilayer membrane in which the hydrophilic regions are in contact with the surrounding solvent, and the hydrophobic regions stay in the center (van Meer, Voelker, & Feigenson, 2008). This structure allows compartmentalization and only appears in aqueous solutions and in the presence of more than one molecule. The plasma membranes of cells follow this pattern of self‐assembly by forming a phospholipid bilayer that delimits the entire cell (Figure 1a). The consequences of these simple interactions are very important and relevant even for the origin of life; this property of self‐assembly of phospholipids is likely to have spontaneously given birth to the first protocells after they formed vesicles around nucleic acids and proteins (Deamer, 2017; Deamer & Barchfeld, 1982).

At larger molecular scales, macromolecules also show complex behaviors from relatively simple pairwise interactions. One of the simplest forms is a chain of nucleotides, which on their own have no enzymatic activity but when organized into polymers with specific sequences can acquire enzymatic properties and become ribozymes (Doudna & Cech, 2002). Similarly, amino acids organized into proteins can acquire enzymatic activities. At a higher level, individual proteins frequently form dimers and higher‐order oligomers because they have interfaces that have affinity for each other (Marianayagam, Sunde, & Matthews, 2004). Together, the properties of oligomers confer important regulatory and functional advantages over just having independent individual proteins (Bergendahl & Marsh, 2017). Many ligand binding sites are formed at the interface between interacting copies of the same protein (Abrusán & Marsh, 2018; Figure 1b). An example is the STING protein, which participates in an immunity pathway conserved from anemones to humans. Its ligands bind to the interface between the individual copies of STING and stabilize the conformation that allows downstream signaling (Kranzusch et al., 2015). The monomers alone do not bind to the ligands and therefore cannot participate in the signaling pathway without first dimerizing. Thus, simple rules that dictate interactions of identical molecules can lead to complex and more efficient functions.

Emerging collective behavior also arises in microorganisms. Quorum sensing is a signaling mechanism that allows bacteria to regulate gene expression and coordinate physiological processes in response to fluctuations in cell population density (Miller & Bassler, 2001). Once a certain threshold of cell density is achieved, each cell reacts to each other and new emergent properties arise in the colony (Figure 1c). This allows some bacteria, such as *Bacillus subtilis* (Cohn, 1872; Ehrenberg, 1835), to adopt a new colonial form referred to as a biofilm (van Gestel, Weissing, Kuipers, & Kovács, 2014; Kalamara, Spacapan, Mandic‐Mulec, & Stanley‐Wall, 2018), which benefits from an increased tolerance to antibiotics. Tolerance is a nonheritable phenotype and can arise when extracellular polymeric substance (EPS) acts as a barrier to antibiotic diffusion. Similarly, tolerance can arise by slowing down metabolism, because numerous antimicrobial drugs target metabolic processes that occur during growth (Flemming et al., 2016). Likewise, *Pseudomonas aeruginosa* (Migula, 1900; Schroeter, 1872) colonies produce virulence factors (Smith & Iglewski, 2003, 2003) and *Vibrio harveyi* (Baumann, Baumann, Bang, & Woolkalis, 1980; Johnson & Shunk, 1936) colonies emit bioluminescence (McDougald, Srinivasan, Rice, & Kjelleberg, 2003), all properties that do not exist in the single cell of noncolonial form.

Pairwise interactions can also lead to a complex organization in macroorganisms, following the same principles as for macromolecules and individual cells. For instance, the V formation of flying migratory birds follows a complex pattern (Figure 1d). The V‐shape improves aerodynamics throughout the group since each bird interacts with its neighbors by creating an upward flow behind the tips of its wings, which gives those who follow it an additional elevation (Nathan & Barbosa, 2008) and saves some energy. Another example is schools of fish in which complex patterns of collective behavior emerge in part due to relatively simple pairwise interactions within the group, for instance for swimming speed adjustment (Katz, Tunstrøm, Ioannou, Huepe, & Couzin, 2011).

As exemplified above, emergent properties stem from the interaction of component parts of a system and take on an unexpected function or property without the need for external intervention or a central “decision” component. An interaction between two proteins in a complex might be easy to imagine, but the set of all interactions within an entire cell, or all pairwise species interactions in an ecosystem, quickly becomes very complex. Complex systems like these can more easily be handled and thought of as networks, and these networks have emergent features of their own, which are shared among different levels of organization.

## 3 EMERGENT FEATURES OF NETWORKS

**FIGURE 2:**
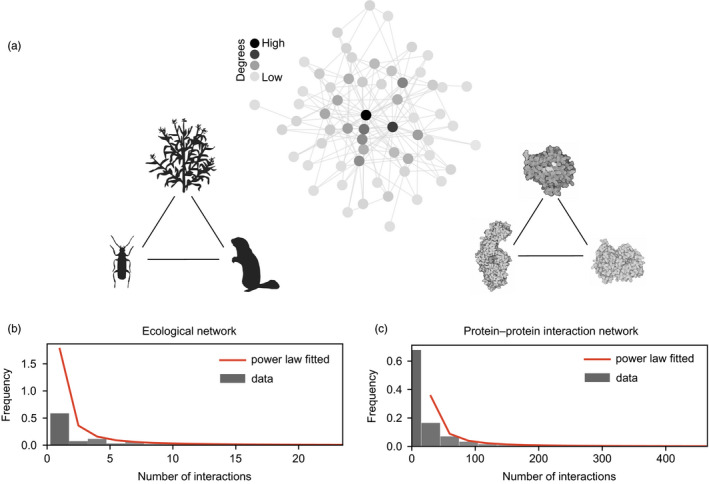
Similar network architectures of ecological and molecular networks. (a) Schematic representation of a scale‐free network. An individual element, or node, of the network (such as a protein in a protein–protein interaction network) is shown as a filled circle. The interactions among the elements are shown as a line (called an edge) connecting the nodes. (b, c) Distributions of number of interactions (also called degrees) in an ecological network and protein–protein interaction network. Distributions are fitted to the power law (P(k) = k^−n^, where k is the number of interactions, P(k) is the fraction of nodes and n is the power law exponent, as shown by the red line. Ecological network data were obtained from Pocock et al study (Pocock et al., 2012). Protein–protein interaction data were retrieved from BioGRID database (Chatr‐Aryamontri et al., 2017). Organism and protein silhouettes were taken from http://phylopic.org

Networks are mathematical objects that represent elements of a system as nodes and their pairwise connections as edges. The comparisons of such networks from different systems have helped identify key emergent properties that define, for instance, how they respond to external changes. Ecological interaction networks (or species interaction networks) are generated by monitoring the interactions (represented by “edges” of the network) between species (represented by “nodes” of the network) (Pocock, Evans, & Memmott, 2012). Interactions between species can be trophic (e.g., predator–prey interactions) or nontrophic (e.g., pollination). By compiling the interactions between pairs of species, a global network can be created (see schematic in Figure 2a). Similarly, protein–protein interaction (PPI) networks are created by monitoring the interactions between pairs of proteins within a cell by various techniques in molecular biology (Miura, 2018). Here too, interactions between proteins can have a positive or negative effect on the activity of the interacting proteins. The ecological and protein–protein interaction networks are well‐studied examples of biological networks. These two types of networks, separated by several organizational levels, remarkably share similarities in their architecture.

Within the framework of networks, proteins and species become analogous elements that can be compared. For instance, the nodes in a network are usually connected to different numbers of partners, which affects the network's properties. In other words, the number of connections (degree) is unevenly distributed among the nodes (Figure 2b,c). A few nodes show high connectedness (hubs), whereas the majority of nodes show limited connectedness.

The degree distribution can be fitted to a power law equation (Figure 2b,c). Networks that follow the power law in this way are called scale‐free networks. A peculiar property of the scale‐free network is that the power law exponent (i.e., the value representing the slope between the log‐scaled frequency of the nodes and corresponding degrees) usually lies between 2 and 3. Interestingly, in networks with such distributions, the removal or perturbation of a randomly selected node is unlikely to perturb the whole network. Indeed, the removal of a highly connected node is usually required to severely perturb a scale‐free network, but this is unlikely since hubs are not very common. These analyses revealed similar properties for networks that from the outset may seem to have no similarity (Barabási & Pósfai, 2016). This property provides inherent robustness to ecological and protein–protein interaction networks and consequently to the corresponding complex systems. From an evolutionary perspective, models suggest that the ways in which genes duplicate and change their interactions through time could be enough to produce such an organization (He & Zhang, 2005; Lynch & Force, 2000).

In order to understand the dynamics of ecological systems, network architecture provides an efficient medium because one can estimate how the removal of a node will likely affect other elements of the network (Delmas et al., 2018). Indeed, several studies show that network topologies (e.g., modularity, nestedness, connectedness) are associated with the robustness of ecosystems (Fortuna et al., 2010). In particular, the stability of ecological systems is of interest to ecologists who can use network‐based approaches in their work (Landi, Minoarivelo, Brännström, Hui, & Dieckmann, 2018). For instance, in some habitats, plant species show high connectedness (Pocock, Evans, & Memmott, 2012). Therefore, the extinction or removal of a plant species in that habitat can presumably have a high impact on the robustness of the system by changing the network topology.

The topology of networks can also be used to understand the robustness of a cell. In some species, genes can be broadly categorized as “essential” or “nonessential” for cell survival. Gene essentiality often correlates with their connectedness in protein–protein interaction networks, in a way similar to the connectedness of important plant species mentioned above. Genes with functions that are essential for survival are more likely to be network hubs than nonessential genes (He & Zhang, 2006). Given the important implications of network architecture, there have been progressive attempts at genome‐wide loss‐of‐function screenings of essential genes through RNAi (Liu et al., 2015) and CRISPR‐Cas9 (Marceau et al., 2016) screens, which confirm that network architecture is a powerful way of representing the relationships among genes.

Overall, these examples illustrate that the robustness of complex systems at the ecological and molecular levels is partly attributable (or at least correlated) to the topological properties of their underlying networks, which themselves depend on rather simple pairwise interactions among their components. System properties therefore transcend levels of organization.

## 4 EVOLUTION OF NOVELTIES IN BIOLOGICAL SYSTEMS

Biological networks are not static throughout evolution. They can change by adding new elements or removing existing ones. In the case of ecological networks, new species can be added by speciation events. One species can diverge into several new ones (Kocher, 2004; Meier et al., 2017), and different species can hybridize and create new species (Eberlein et al., 2019; Leducq et al., 2016; Mavárez et al., 2006; Soltis & Soltis, 2009). These principles present parallels for the emergence of functional proteins. New proteins most often arise from genes that have been duplicated (Aury et al., 2006; Bomblies & Madlung, 2014; Guan, Dunham, & Troyanskaya, 2007; Hakes, Pinney, Lovell, Oliver, & Robertson, 2007; Zhang, 2003). Although their functions may diverge from that of their ancestor, these new proteins often inherit properties from them, including their interactions with other proteins. Similarly, new species can inherit properties from their progenitors, like their trophic relationships with other species. In this section, we will discuss the processes by which novel elements appear at different organization levels.

Gene duplication produces an identical copy of a preexisting gene, usually through errors in DNA replication, cell division, unequal crossing over during recombination, or retrotransposition events (Otto & Whitton, 2000; Reams & Roth, 2015; Zhang, 2003). The duplicates, or paralogs, are identical at first, but they diverge as they accumulate mutations. As a result, they can divide the ancestral gene's functions so that they can be separately optimized (Baker, Hanson‐Smith, & Johnson, 2013; Force et al., 1999; Lambert, Cochran, Wilde, Olsen, & Cooper, 2015; Lynch & Conery, 2000) or develop new functions (Boncoeur et al., 2012; Bridgham, Brown, Rodríguez‐Marí, Catchen, & Thornton, 2008; Lien et al., 2016; Sandve, Rohlfs, & Hvidsten, 2018). The relative ease with which a gene can be duplicated, and the possibilities for functional divergence make gene duplication one of the main drivers of functional innovation in the genome. These changes at the molecular level can have direct ecological consequences. For example, Antarctic zoarcids evolved efficient freeze resistance after a gene duplication event, allowing them to survive in a cold environment. This novel freeze resistance protein derived from a bifunctional ancestral protein that could both synthesize sialic acid and bind to ice crystals to prevent their growth. After the duplication, one of the copies lost several exons and acquired mutations that allowed for the optimization of the antifreeze activity (Deng, Cheng, Ye, He, & Chen, 2010).

**FIGURE 3:**
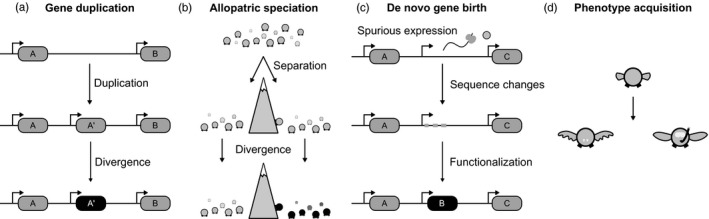
Mechanisms that generate novelties at the genetic and ecological levels. Gene duplication (a) and allopatric speciation (b) generate novelties from preexisting elements. (a) The new copy of the gene is initially identical to its progenitor, but it can diverge with time and acquire new functions. (b) In allopatric speciation, a population is physically separated, which leads to divergence and the potential development of a new species. De novo gene birth (c) and phenotype acquisition (d) generate unexpected novelties. (c) In de novo gene birth, pervasive translation of an intergenic sequence can expose the sequence to purifying selection. However, specific mutations may confer a beneficial function, in which case it becomes a new gene. (d) In the case of phenotype acquisition, anatomical structures can evolve from structures that were nonfunctional or served a different purpose in their ancestors

The way gene duplication paves the way for new functions has some analogy with speciation by allopatry, that is, speciation as a consequence of physical separation (Figure 3; Mayr, 1942, 1947). In this classical mechanism of speciation, a population is separated into two groups by a change in the environment, such as the appearance of a mountain range (Barrera‐Guzmán, Milá, Sánchez‐González, & Navarro‐Sigüenza, 2012; Chaves & Smith, 2011; Gutiérrez‐Pinto et al., 2012), or in more recent history, a highway (Thompson, Rieseberg, & Schluter, 2018). While the two groups would be very similar at first, the separation prevents gene flow. Through time, random mutations and recombination would lead to the appearance of different variants in each of the two groups. In fact, they could face selection on different traits, and develop different phenotypes (Barrett et al., 2019) or even become separate species by reproductive isolation, due to, for example, the incompatibility between the gene and protein networks of the two species (Dobzhansky, 1934; Mayr, 1942, 1947; Muller, 1942). Similarly, after gene duplication the two paralogs are identical at first, but can accumulate separate mutations (Force et al., 1999). Indeed, paralogs might face selection for different properties, as in the case of the freeze resistance proteins discussed above (Deng et al., 2010). One of the limitations of this analogy between speciation and gene duplication is that, while gene flow is limited between the allopatric populations, paralogs recombine with each other with varying frequencies (depending on the locus), and can sometimes transfer variants unilaterally through gene conversion (Chen, Cooper, Chuzhanova, Férec, & Patrinos, 2007; Dumont, 2015). Nevertheless, paralogs can reach a level of sequence divergence that allows them to minimize recombination (Harpak, Lan, Gao, & Pritchard, 2017), reminiscent of how species become reproductively isolated.

As is the case for new species, the relationships between paralogs can become complex with time. Paralogs are often redundant and can compensate for each other's loss to a certain degree (DeLuna et al., 2008; Gagnon‐Arsenault et al., 2013; Hsiao & Vitkup, 2008), thereby contributing to the robustness of cellular networks (Diss et al., 2014). This behavior can also be seen at the community level in ecological equivalents, that is, organisms that serve similar functions in different communities (Hubbell, 2006; Lincoln, 1998). Some ecological equivalents, like Mustela nigripes (black‐footed ferrets) (Audubon & Bachman, 1851) and Mustela eversmanii (Siberian polecats) (Lesson, 1827), can at least partially replace the other species in their communities (Biggins, Hanebury, Miller, & Powell, 2011), similar to how some genes can compensate for the loss of their paralog.

However, despite their inherent similarity, not all paralogs are able to compensate for each other. In fact, some proteins need to interact with their paralog to function correctly (Baker et al., 2013; Boncoeur et al., 2012; Bridgham et al., 2008; DeLuna, Springer, Kirschner, & Kishony, 2010; Diss et al., 2017). Interestingly, similar relationships are also seen at an ecological level with closely related species that become mutualistic. This is the case for the cichlid species *Etroplus maculatus* (Bloch, 1795) and *Etroplus suratensis* (Bloch, 1790) that evolved a cleaning symbiosis (Wyman & Ward, 1972), the cichlid species *Archocentrus nigrofasciatus* (Günther, 1867) that benefits from *Hypsophrys nicaraguensis* (Günther, 1864) to avoid predation (Lehtonen, 2008), and the ant species *Dolichoderus debilis* (Emery, 1890) and *Crematogaster levior* (Longino, 2003) that share nests and forage together (Forel, 1898; Jarau & Hrncir, 2009). Although closely related, these duplicated genes or species cannot replace one another because they depend on each other. How these relationships evolved is still being investigated, but a necessary condition for evolving them is to maintain the interaction between the proteins or species. Research suggests that keeping similar traits increases the chance of maintaining the interactions. In the cichlids example, the cleaned species mimics a posture shown by adult members of the other species, which prompts the cleaning activity (Wyman & Ward, 1972). As for the ant species, they secrete similar molecules, which allow for the recognition of their ally and keep them from attacking each other (Menzel et al., 2013; Menzel, Linsenmair, & Blüthgen, 2008). Finally, a higher overall protein sequence similarity between paralogs favors the retention of their interactions with the same partners, including with one another (Ispolatov, Yuryev, Mazo, & Maslov, 2005; Lukatsky, Shakhnovich, Mintseris, & Shakhnovich, 2007; Lukatsky, Zeldovich, & Shakhnovich, 2006; Marchant et al., 2019).

Just like speciation and gene duplication, phenotypic changes arise through the modification of preexisting material. Novel phenotypes allow organisms to fill unoccupied niches; for example, mammals have taken to both the sea and the sky over the course of their evolution. To our eyes in the present day, their innovation is apparent and can even be species defining. For example, bats’ wings and whales’ fins share ancestry with the limbs of quadrupedal animals (Cooper & Tabin, 2008; Teeling et al., 2005; Thewissen, Cooper, George, & Bajpai, 2009), but now they match the performance of other limbs that serve similar functions in their environments, such as bird wings and fish fins. In the genome, new genes can also emerge from unexpected places (Figure 3). In the first decade of this century, genes that had evolved from previously nongenic sequences were described (de novo genes) (Begun, Lindfors, Kern, & Jones, 2007; Levine, Jones, Kern, Lindfors, & Begun, 2006). This was a major shift in the field of novel genes, as de novo genes were deemed practically impossible until the 21st century (Tautz, 2014). Since then, de novo genes have been found in the genomes of many different organisms, from yeast (Cai, Zhao, Jiang, & Wang, 2008) to primates (Toll‐Riera et al., 2009) to plants (Zhang et al., 2019).

It can be difficult to understand how some of these phenotypes arose. What use is an eye before it is fully functional? What use is a wing if it cannot be used for flight? However, these are cases where rudimentary forms of the phenotype yield a selective advantage; eyes that cannot form images can still sense light differences (Lamb, Collin, & Pugh, 2007), and wings that cannot propel can still be used for a controlled descent (Kaplan, 2011). Having meaningful intermediate states is required for the evolution of organismal traits as it is for the evolution of protein sequences and function (Smith, 1970). Unfortunately, the picture is not yet as clear with de novo genes; the mechanisms by which a new gene can emerge from noncoding regions are not fully understood. It is difficult to envision how a random DNA sequence that is expressed into a protein could have anything but a negative fitness effect. However, an elegant solution to this problem is found in the mounting evidence that a majority of the genome is transcribed, if at low levels (Clark, Amaral, Schlesinger, & Mattick, 2011; Havilio, Levanon, Lerman, Kupiec, & Eisenberg, 2005; Kapranov et al., 2002). In fact, it has been shown that large parts of the “noncoding” genome are translated at low levels (Ji, Song, Regev, & Struhl, 2015; Ruiz‐Orera, Verdaguer‐Grau, Villanueva‐Cañas, Messeguer, & Mar Albà, 2018; Wilson & Masel, 2011). This pervasive transcription and translation allow intergenic regions to be molded by natural selection and increase their probability to gain a function in the cell (Wilson & Masel, 2011).

## 5 CONFLICTS IN CELLULAR SYSTEMS AND IN THE ENVIRONMENT

Evolutionary novelties are often retained if they confer advantages to an organism. However, an advantage for one organism might result in a disadvantage for another one, which leads to conflict. Conflicts may occur when the interests of two or more interacting parties drive their fitness in opposite directions (Queller, 2014). Although the term conflict primarily derives from ecology, it has been extensively adopted in molecular biology to describe interactions between fragments of genetic material and biomolecules. Interactions can be both between‐species and within‐species, and between‐cells and within‐cells. The only requirement for an element to trigger the conflict is to be able to replicate and to have heritable variation, which means that it applies to genes and organisms. One reason why conflicts deserve attention is that they can stimulate the evolution of complex molecular and organismal traits that would otherwise not have evolved. Indeed, a stable equilibrium is rarely established, which often leads to the constant evolution of traits, new adaptations, increased diversity, and eventually the evolution of more complex systems.

## 6 INTERSPECIFIC CONFLICT

A conflict between species typically includes interactions between prey and predator, or host and parasite. As formalized in the Red Queen hypothesis (Van Valen, 1973), the antagonistic pressures exerted by the interacting parties on each other accelerate their evolution, while retaining a stable fitness. Such conflict‐driven evolution can manifest in different ways, for example, by improving the ability to recognize the opponent, by developing “weapons” to fight it, by confusing it through mimicry, and many others (Queller & Strassmann, 2018). Prey–predator dynamics can affect sensory systems, behavior, and life‐history traits of both species to improve each other's recognition, as it is in the case of bats and moths; bats use an echolocation system to track moths, whereas moths have developed sensory organs to hear bat calls (Ter Hofstede & Ratcliffe, 2016). At the cellular level, in a conflict between a host and pathogen, proteins of different organisms evolve to recognize each other. Organisms have developed systems to detect foreign cells and molecules that depend on the binding abilities of macromolecules such as proteins. Host proteins affected by pathogens (“host factors”) determine the strength of infection based on the underlying network of interacting proteins. Pathogens usually target highly connected proteins, which leads to perturbation of many cellular functions and more severe consequences for an organism. Specifically, viruses target host proteins that can connect to any other protein in the network via a small number of intermediate interactions (Liu et al., 2015; Wuchty, 2014). In this way, pathogens and parasites use the network properties of cell hosts to their advantage.

Accelerated evolution caused by conflicts can be detected by the elevated rate of protein evolution (positive selection). This phenomenon has been observed at the binding sites of interacting proteins (McLaughlin & Malik, 2017). For instance, in mammals, two paralogs, TRIM5a and TRIM22, recognize retroviral proteins. Since retroviruses evolve rapidly, these proteins are expected to also evolve rapidly to maintain their ability to recognize viral proteins. Positive selection within the two domains of each protein responsible for antiviral specificity was shown to accelerate their evolution, revealing that the conflict may accelerate the divergence of paralogous proteins (Sawyer, Emerman, & Malik, 2007). Finally, human proteins that interact with viruses were shown to, on average, evolve under stronger positive selection than those that do not interact with viruses (Enard, Cai, Gwennap, & Petrov, 2016).

**FIGURE 4:**
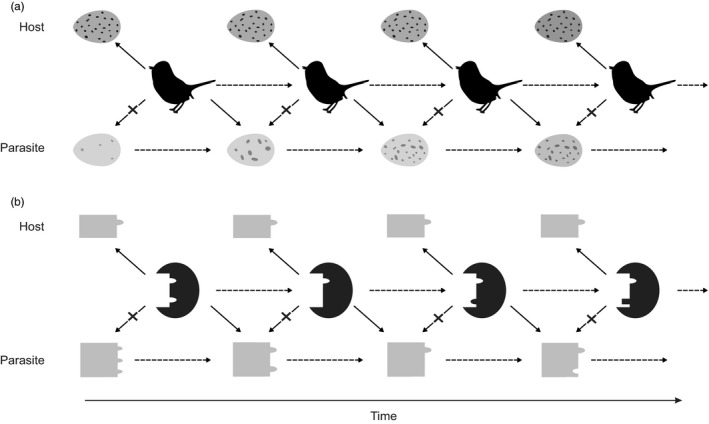
Mimicry evolving from interspecific conflict has similar features at different levels of organization. (a) Birds do not experience fitness costs if they are able to distinguish their own eggs (top row of eggs) from the parasitic eggs of cuckoo (bottom row of eggs). Cuckoo eggs evolve to mimic the host egg, making birds unable to distinguish and remove the parasitic eggs. In turn, hosts evolve improved recognition of mimic eggs. (b) Host's protein kinase R (PKR) triggers an immune response in primates upon binding to eIF2alpha (top row), causing cell growth arrest, and limiting production of virus particles. Virus protein K3L mimics eIF2alpha and binds to PKR, blocking its function. The binding surface of PKR evolves to recognize the virus while at the same time retaining the ability to detect the conserved structure of eIF2alpha. In response, virus proteins evolve to better mimic eIF2alpha. Figure inspired by McLaughlin & Malik (2017). Bird silhouette was taken from http://phylopic.org

Mimicry is a strategy that escalates conflicts both at the molecular and organismal levels. A mimic evolves to resemble its opponent or opponent's components (e.g., eggs or proteins) and evade recognition. This is famously exemplified by the common cuckoo, *Cuculus canorus* (Linnaeus, 1758), which lays its eggs in other species' nests, removes host eggs, and parasitizes upon the parental care of the host species. The cuckoo's egg color and pattern evolve to match those of the host eggs, whereas the host bird sensory system evolves to better recognize parasitic eggs (Stoddard & Stevens, 2011). Similar coevolutionary patterns can be observed at a much smaller scale, for instance, between host cells and their pathogens. K3L is a rapidly evolving protein in poxviruses that mimics and competes with the primate protein eIF2alpha. elF2alpha is a component of the immune system that triggers an immune response when bound by the host's protein kinase R, suppressing virus growth (Elde, Child, Geballe, & Malik, 2009). The virus improves the resemblance of K3L to eIF2alpha in order for K3L to be bound by protein kinase R instead, abolishing the immune response. However, the host's protein kinase R in turn evolves to avoid binding to the virus protein while still binding to the conserved native one (Figure 4).

Interspecific conflict often leads to the evolution of new adaptations, such as weapons, behaviors, or protective structures. Toxin and antitoxin production is a common strategy of defense or attack, from vertebrates (Hanifin, Brodie, & Brodie, 2008) to insects and microbes (Pedrini et al., 2015). Parasites usually evolve refined adaptations to lure and infect their host and complete their reproductive cycle, which includes changing the behavior of the attacked host (de Bekker et al., 2014; Dubey, 2014). Fungal pathogens, for instance, present a large repertoire of shields, weapons, structures, and strategies during infection to avoid phagocytosis (Erwig & Gow, 2016). *Candida albicans* (C.P. Robin; Berkhout, 1923) grows elongated hyphae, whereas *Cryptococcus neoformans* (Sanfelice; Vuillemin, 1901) can undergo morphological transition into enormous Titan cells during infection (Zaragoza & Nielsen, 2013) as a means to avoid engulfment by phagocytes.

## 7 INTRACELLULAR CONFLICTS

Conflicts do not only exist between cells and organisms, but also among the genes encoded in the genome of a single cell. Just like interactions between species are central to the definition of an ecosystem (Willis, 1997), interactions between genes in a genome are essential for the function of the cell (Tarassov et al., 2008). While there is little doubt that a large proportion of these interactions evolved because they were adaptive, some exist only to cope with genetic conflicts. Conflicts among genes often arise because the inheritance mode of some genes violates one or several of the principles of Mendelian inheritance (Werren, 2011). This raises the possibility that such a gene would be inherited more often than expected and thus have higher fitness. Natural selection would act on the gene itself to favor its spread, which could be associated with a decrease in the fitness of the host. As a result, the host and the selfish gene will initiate an evolutionary arms race, analogous to those occurring at the intercellular level.

**FIGURE 5:**
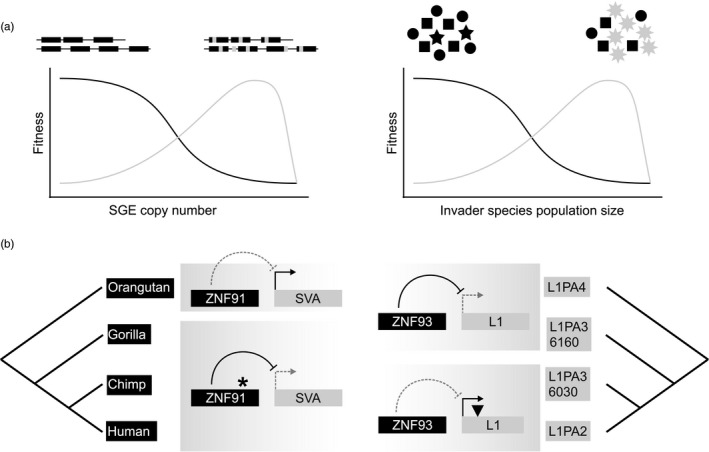
Conflicts within genomes and ecosystems. (a) Conflict between the host genome and selfish genetic elements (SGEs, left) or between native and invader species (right) can be schematically described by similar fitness functions. In both cases, the “host” (black) is advantaged when the “invader” (grey) is at low frequency. Inversely, the “invader” gains fitness with higher frequency. In both cases, we can imagine that very high SGE copy numbers or large invader population sizes can drive the extinction of the host species or disruption of the native ecosystem, respectively. Figure inspired by Queller and Strassmann (2018). (b) The conflict between retrotransposons and host transcription repressors in hominids led to mutations (star and triangle symbols) accumulating in a manner consistent with an arms race. Left: The ZNF91 KRAB zinc finger protein acquired mutations in the common ancestor of gorilla, chimp, and humans that allow it to repress the activity of the SVA retrotransposon. Right: Subfamilies of L1 retrotransposons acquired a mutation that allows them to escape repression mediated by ZNF93

The selfish spread of a gene can be compared to the spread of invasive species in an ecosystem, which is largely disconnected from the fitness of the other species in the ecosystem (Figure 5a) and may eventually jeopardize the maintenance of the ecosystem itself. Models of ecological networks that integrate trophic interactions between species predicted that some properties of species, like generalism, can predict their chances of success in an invasion (Romanuk et al., 2009). Thus, non‐Mendelian inheritance is a key property within a genome that predicts the selfish spread of a gene, while ecological properties can predict species invasiveness at the ecosystem level. Conflicts impose a selective pressure on “host” components (genes in a genome or species in an ecosystem) to evolve mechanisms that limit the spread of the “selfish” genes or species. Adaptive evolution to invaders within ecosystems can involve various classes of traits, spanning morphological, physiological, behavioral, and life‐history traits (Strauss, Lau, & Carroll, 2006). Similarly, selfish genetic elements pressure host genes to evolve repression mechanisms, as exemplified below. In this way, one can imagine both genomes and ecosystems as coherent systems harboring some interactions that may only serve to buffer the detrimental effects caused by their cheating components.

One type of non‐Mendelian transmission pattern that can lead to conflict is when chromosomes do not segregate in equal ratios to the gametes during meiosis (Werren, 2011). One of several mechanisms that can cause this relies on the asymmetry of female meiosis. Only one out of four meiotic products becomes a gamete during female meiosis, enabling the rise of selfish chromosomes that increase their odds of being transmitted to the oocyte (Henikoff, Ahmad, & Malik, 2001). Chromosomes that exhibit such a segregation distortion exploit the meiotic spindle machinery to favor their inheritance to the oocyte (Akera et al., 2017; Hewitt, 1976). This distortion motivated the elegant centromere drive hypothesis (Henikoff et al., 2001), which posits that a conflict exists between female and male meiosis. The evolution of “stronger” centromeres in asymmetrical female meiosis would be deleterious in male meiosis, which is symmetrical and might suffer more from nondisjunction. Empirical evidence of the unexpectedly fast and adaptive evolution of centromeric DNA repeats and histone proteins, respectively (Kursel & Malik, 2018), are remarkably consistent with an arms race resulting from this conflict.

Centromere drive is one among many types of meiotic drive. Another type is caused by killer meiotic drivers, which selectively eliminate meiotic products that do not harbor the drive element or that contain the target locus conferring killer sensitivity (Bravo Núñez, Nuckolls, & Zanders, 2018). Spore killing in some ascomycete fungi (Turner & Perkins, 1991) and segregation distorters in *Drosophila* (Larracuente & Presgraves, 2012) are examples of drive systems in which gametes harboring the killer locus eliminate sensitive gametes before they can develop or fertilize other ones. These examples of male meiotic drive bear a certain analogy with siblicide. As an extreme form of sibling conflict, siblicide has been documented in many species (Furness, Morrison, Orr, Arendt, & Reznick, 2015). For instance, in many bird species, individuals within a brood can cause the death of their siblings, either because of aggressive behavior or because of competition for food (Mock, 1984; Redondo, Romero, Díaz‐Delgado, & Nagy, 2019).

Another type of deviation from Mendelian transmission is the spread of transposable elements (TEs) within genomes. If we consider the completion of a single cell cycle through a mitotic division, each canonical gene on each chromosome of a given genome is replicated exactly once, and both resulting cells inherit one copy of this gene. In contrast, TEs can replicate more than once within a single cell cycle, with copies of themselves invading new loci on chromosomes (McClintock, 1950). It soon appeared clear that like all mutation types, TEs mostly have deleterious effects upon insertion into the host genome, as illustrated by the classic example of TEs contributing to hybrid dysgenesis in *Drosophila* (Rubin, Kidwell, & Bingham, 1982). Population genetic models also support a scenario in which selection against transposition is the main determinant of TE frequency patterns in natural populations (Charlesworth, Sniegowski, & Stephan, 1994).

TEs will often encode genes that are necessary but not sufficient for their own replication. For example, retrotransposons encode genes that are essential for their own reverse transcription and integration into the host genome (Wicker et al., 2007). However, the initial production of retrotransposon mRNAs relies on the host transcription machinery. Thus, a conflict can arise from the host's need to express its own genes and the retrotransposon's need to replicate itself. Starting from an equilibrium state between retrotransposon expansion and repression from the host, we can imagine that a retrotransposon mutant favoring its own replication would pressure host genes to evolve stronger repression in response to the deleterious effects of a retrotransposition expansion. In humans, this hypothetical scenario is illustrated by the conflict between some retrotransposon subfamilies and KRAB zinc finger proteins that bind dsDNA and recruit a transcriptional repressor complex. It was shown that the human zinc finger protein ZNF91 is efficient in silencing SVA retrotransposons, while a reconstructed ancestral ZNF91 gene sequence is not (Jacobs et al., 2014) (Figure 5b, left). This repression likely imposes a cost on the host, as it also abrogates the transcription of host genes in the vicinity of SVA insertions. TE repression imposing a cost by repressing neighboring host genes is also hypothesized in the case of TE methylation in *Arabidopsis*, as regions near genes are deprived in methylated TEs (Ahmed, Sarazin, Bowler, Colot, & Quesneville, 2011). Additionally, the repressing ability of the related human zinc finger protein ZNF93 against a subfamily of L1 retrotransposons was counteracted by a deletion in the ZNF93 binding site of the L1 sequence (Jacobs et al., 2014) (Figure 5b, right). These lines of evidence support the hypothesis of an arms race taking place between KRAB zinc finger proteins and retrotransposons in hominid evolution.

In summary, arms races and conflicts are common themes that emerge from the comparison of selfish components within genomes and ecosystems. Thus, it can be expected that these conflicts lead to the emergence of complex systems that cannot be explained by adaptive evolution of host features alone, but rather by compensatory evolution in the face of cheaters.

## 8 DISCUSSION/CONCLUSION

Molecular biology and ecology have analogous properties that can be exploited in teaching and research. We expanded on these ideas by discussing how molecular phenomena closely resemble what is seen at the organismal level. These parallels call for deeper integration of the fields since models from one field can inspire models for analogous processes in the other. For example, networks can be used to represent interactions between molecules and between species equally well; new functions and species can emerge from modification of the existing ones; and arms races have been described at the species and molecular levels. While we focused on these examples that are close to our fields of research, there are potentially many more.

Having concepts carry over between fields allows models to be transferable. As described above, networks have been used at both the molecular and ecological levels. The well‐established framework of networks has contributed to the advancement of both fields to solve analogous problems, such as the identification of genes involved in diseases (Yu, Kim, Xiao, & Hwang, 2013) and species critical to ecosystem conservation (Harvey, Gounand, Ward, & Altermatt, 2017) based on their positions and connectedness in networks. These models of conservation benefit from the comparison and the principles of stoichiometry. Proteins that are highly connected in a network are also highly abundant (Saeed & Deane, 2006), just like the optimal population sizes of a given species in an ecosystem depend on their specific roles and their interactions with other species (Harvey et al., 2017; Sterner & Elser, 2002). Elsewhere, the study of gene duplication has benefitted from comparisons to speciation. For instance, models on how several species emerge from an ancestral one (adaptive radiation) have been used to describe how gene families emerge by gene duplication. While this model's predictions match well the evolution of olfactory receptor families (Francino, 2005), further research could provide more information on how new functions are acquired through gene duplication. Ultimately, these cases show how our understanding of specific processes can benefit from models based on analogous phenomena.

Traditionally, molecular biology and ecology are taught separately as if they were somehow disconnected. Considering the similarities between molecular biology and ecology, a case can be made for a more integrative approach in teaching. This kind of organization could potentially lead to advancement of both fields by bringing students to think about the overarching properties of biological systems. We believe that students’ curricula and activities could be modified to facilitate better communication between these fields and to focus on commonalities among systems rather than on the differences. From a pedagogical point of view, narratives from one level can be borrowed to help teach key concepts at other levels, depending on the training of the people involved. As a result, future professionals and researchers would be able to come up with original ideas to study and understand nature better as a whole.

## Data Availability

Data sharing is not applicable to this article as no new data were created or analyzed in this study.

## References

[eva12961-bib-0001] Abrusán, G. , & Marsh, J. A. (2018). Ligand binding site structure influences the evolution of protein complex function and topology. Cell Reports, 22(12), 3265–3276. 10.1016/j.celrep.2018.02.085 29562182 PMC5873459

[eva12961-bib-0002] Ahmed, I. , Sarazin, A. , Bowler, C. , Colot, V. , & Quesneville, H. (2011). Genome‐wide evidence for local DNA methylation spreading from small RNA‐targeted sequences in Arabidopsis. Nucleic Acids Research, 39(16), 6919–6931. 10.1093/nar/gkr324 21586580 PMC3167636

[eva12961-bib-0003] Akera, T. , Chmátal, L. , Trimm, E. , Yang, K. , Aonbangkhen, C. , Chenoweth, D. M. , … Lampson, M. A. (2017). Spindle asymmetry drives non‐Mendelian chromosome segregation. Science, 358(6363), 668–672. 29097549 10.1126/science.aan0092 PMC5906099

[eva12961-bib-0004] Audubon, J. J. , & Bachman, J. (1851). The Quadrupeds of North America. New York, NY: V.G. Audubon.

[eva12961-bib-0005] Aury, J.‐M. , Jaillon, O. , Duret, L. , Noel, B. , Jubin, C. , Porcel, B. M. , … Wincker, P. (2006). Global trends of whole‐genome duplications revealed by the ciliate Paramecium tetraurelia. Nature, 444(7116), 171–178. 17086204 10.1038/nature05230

[eva12961-bib-0006] Avise, J. C. (2001). Evolving genomic metaphors: A new look at the language of DNA. Science, 294(5540), 86–87. 11588247 10.1126/science.294.5540.86

[eva12961-bib-0007] Baker, C. R. , Hanson‐Smith, V. , & Johnson, A. D. (2013). Following gene duplication, paralog interference constrains transcriptional circuit evolution. Science, 342(6154), 104–108. 24092741 10.1126/science.1240810 PMC3911913

[eva12961-bib-0008] Barabási, A.‐L. , & Pósfai, M. (2016). Network Science. Cambridge: Cambridge University Press.

[eva12961-bib-0009] Barrera‐Guzmán, A. O. , Milá, B. , Sánchez‐González, L. A. , & Navarro‐Sigüenza, A. G. (2012). Speciation in an avian complex endemic to the mountains of Middle America (Ergaticus, Aves: Parulidae). Molecular Phylogenetics and Evolution, 62(3), 907–920. 10.1016/j.ympev.2011.11.020 22155712

[eva12961-bib-0010] Barrett, R. D. H. , Laurent, S. , Mallarino, R. , Pfeifer, S. P. , Xu, C. C. Y. , Foll, M. , … Hoekstra, H. E. (2019). Linking a mutation to survival in wild mice. Science, 363(6426), 499–504. 30705186 10.1126/science.aav3824

[eva12961-bib-0011] Baumann, P. , Baumann, L. , Bang, S. S. , & Woolkalis, M. J. (1980). Reevaluation of the taxonomy of Vibrio, Beneckea, and Photobacterium: Abolition of the genus Beneckea. Current Microbiology, 4(3), 127–132.

[eva12961-bib-0012] Begun, D. J. , Lindfors, H. A. , Kern, A. D. , & Jones, C. D. (2007). Evidence for de novo evolution of testis‐expressed genes in the Drosophila yakuba/Drosophila erecta clade. Genetics, 176(2), 1131–1137. 10.1534/genetics.106.069245 17435230 PMC1894579

[eva12961-bib-0013] Bergendahl, L. T. , & Marsh, J. A. (2017). Functional determinants of protein assembly into homomeric complexes. Scientific Reports, 7(1), 4932. 28694495 10.1038/s41598-017-05084-8 PMC5504011

[eva12961-bib-0014] Berkhout, C. M. (1923). De Schimmelgeslachten Monilia, Oidium. Edauw en Johannissen: Oospora en Torula.

[eva12961-bib-0015] Biggins, D. E. , Hanebury, L. R. , Miller, B. J. , & Powell, R. A. (2011). Black‐footed ferrets and Siberian polecats as ecological surrogates and ecological equivalents. Journal of Mammalogy, 92(4), 710–720. 10.1644/10-MAMM-S-110.1

[eva12961-bib-0016] Bloch, M. E. (1790). Naturgeschichte der ausländischen Fische (Vol. 4). Verfasser.

[eva12961-bib-0017] Bloch, M. E. (1795). Naturgeschichte der ausländischen Fische (Vol. 9). Verfasser.

[eva12961-bib-0018] Bomblies, K. , & Madlung, A. (2014). Polyploidy in the Arabidopsis genus. Chromosome Research, 22(2), 117–134. 10.1007/s10577-014-9416-x 24788061

[eva12961-bib-0019] Boncoeur, E. , Durmort, C. , Bernay, B. , Ebel, C. , Di Guilmi, A. M. , Croizé, J. , … Jault, J.‐M. (2012). PatA and PatB form a functional heterodimeric ABC multidrug efflux transporter responsible for the resistance of Streptococcus pneumoniae to fluoroquinolones. Biochemistry, 51(39), 7755–7765. 10.1021/bi300762p 22950454

[eva12961-bib-0020] Bravo Núñez, M. A. , Nuckolls, N. L. , & Zanders, S. E. (2018). Genetic villains: Killer meiotic drivers. Trends in Genetics: TIG, 34(6), 424–433. 10.1016/j.tig.2018.02.003 29499907 PMC5959745

[eva12961-bib-0021] Bridgham, J. T. , Brown, J. E. , Rodríguez‐Marí, A. , Catchen, J. M. , & Thornton, J. W. (2008). Evolution of a new function by degenerative mutation in cephalochordate steroid receptors. PLoS Genetics, 4(9), e1000191 10.1371/journal.pgen.1000191 18787702 PMC2527136

[eva12961-bib-0022] Cai, J. , Zhao, R. , Jiang, H. , & Wang, W. (2008). De novo origination of a new protein‐coding gene in *Saccharomyces cerevisiae* . Genetics, 179(1), 487–496. 10.1534/genetics.107.084491 18493065 PMC2390625

[eva12961-bib-0023] Charlesworth, B. , Sniegowski, P. , & Stephan, W. (1994). The evolutionary dynamics of repetitive DNA in eukaryotes. Nature, 371(6494), 215–220. 10.1038/371215a0 8078581

[eva12961-bib-0024] Chatr‐Aryamontri, A. , Oughtred, R. , Boucher, L. , Rust, J. , Chang, C. , Kolas, N. K. , … Tyers, M. (2017). The BioGRID interaction database: 2017 update. Nucleic Acids Research, 45(D1), D369–D379. 10.1093/nar/gkw1102 27980099 PMC5210573

[eva12961-bib-0025] Chaves, J. A. , & Smith, T. B. (2011). Evolutionary patterns of diversification in the Andean hummingbird genus Adelomyia. Molecular Phylogenetics and Evolution, 60(2), 207–218. 10.1016/j.ympev.2011.04.007 21558009

[eva12961-bib-0026] Chen, J.‐M. , Cooper, D. N. , Chuzhanova, N. , Férec, C. , & Patrinos, G. P. (2007). Gene conversion: Mechanisms, evolution and human disease. Nature Reviews Genetics, 8(10), 762–775. 10.1038/nrg2193 17846636

[eva12961-bib-0027] Clark, M. B. , Amaral, P. P. , Schlesinger, F. J. , … Mattick, J. S. (2011). The reality of pervasive transcription. PLoS Biology, 9(7), e1000625 10.1371/journal.pbio.1000625 21765801 PMC3134446

[eva12961-bib-0028] Cohn, F. (1872). Untersuchungen über Bakterien. Beitrage Zur Biologie Der Pflanzen., 1(2), 127–224.

[eva12961-bib-0029] Cooper, K. L. , & Tabin, C. J. (2008). Understanding of bat wing evolution takes flight. Genes & Development, 22(2), 121–124. 10.1101/gad.1639108 18198331 PMC2731632

[eva12961-bib-0030] de Bekker, C. , Quevillon, L. E. , Smith, P. B. , Fleming, K. R. , Ghosh, D. , Patterson, A. D. , & Hughes, D. P. (2014). Species‐specific ant brain manipulation by a specialized fungal parasite. BMC Evolutionary Biology, 14, 166 10.1186/s12862-014-0166-3 25085339 PMC4174324

[eva12961-bib-0031] Deamer, D. W. (2017). The role of lipid membranes in life’s origin. Life, 7(1), 5 10.3390/life7010005 PMC5370405 28106741

[eva12961-bib-0032] Deamer, D. W. , & Barchfeld, G. L. (1982). Encapsulation of macromolecules by lipid vesicles under simulated prebiotic conditions. Journal of Molecular Evolution, 18(3), 203–206. 10.1007/BF01733047 7097780

[eva12961-bib-0033] Delmas, E. , Besson, M. , Brice, M.‐H. , Burkle, L. A. , Dalla Riva, G. V. , Fortin, M.‐J. , … Poisot, T. (2018). Analysing ecological networks of species interactions. Biological Reviews of the Cambridge Philosophical Society, 10.1111/brv.12433 29923657

[eva12961-bib-0034] DeLuna, A. , Springer, M. , Kirschner, M. W. , & Kishony, R. (2010). Need‐based up‐regulation of protein levels in response to deletion of their duplicate genes. PLoS Biology, 8(3), e1000347 10.1371/journal.pbio.1000347 20361019 PMC2846854

[eva12961-bib-0035] DeLuna, A. , Vetsigian, K. , Shoresh, N. , Hegreness, M. , Colón‐González, M. , Chao, S. , & Kishony, R. (2008). Exposing the fitness contribution of duplicated genes. Nature Genetics, 40(5), 676–681. 10.1038/ng.123 18408719

[eva12961-bib-0036] Deng, C. , Cheng, C.‐H.‐C. , Ye, H. , He, X. , & Chen, L. (2010). Evolution of an antifreeze protein by neofunctionalization under escape from adaptive conflict. Proceedings of the National Academy of Sciences of the United States of America, 107(50), 21593–21598. 10.1073/pnas.1007883107 21115821 PMC3003108

[eva12961-bib-0037] Diss, G. , Ascencio, D. , DeLuna, A. , & Landry, C. R. (2014). Molecular mechanisms of paralogous compensation and the robustness of cellular networks. Journal of Experimental Zoology. Part B, Molecular and Developmental Evolution, 322(7), 488–499. 10.1002/jez.b.22555 24376223

[eva12961-bib-0038] Diss, G. , Gagnon‐Arsenault, I. , Dion‐Coté, A.‐M. , Vignaud, H. , Ascencio, D. I. , Berger, C. M. , & Landry, C. R. (2017). Gene duplication can impart fragility, not robustness, in the yeast protein interaction network. Science, 355(6325), 630–634. 10.1126/science.aai7685 28183979

[eva12961-bib-0039] Dobzhansky, T. (1934). Studies on hybrid sterility. Zeitschrift Für Zellforschung Und Mikroskopische Anatomie, 21(2), 169–223. 10.1007/BF00374056

[eva12961-bib-0040] Doudna, J. A. , & Cech, T. R. (2002). The chemical repertoire of natural ribozymes. Nature, 418(6894), 222–228. 10.1038/418222a 12110898

[eva12961-bib-0041] Dubey, J. P. . (2014). Chapter 1 – The history and life cycle of *Toxoplasma gondii* In Weiss L. M., & Kim K. (Eds.), Toxoplasma gondii (2nd Edition, pp. 1–17). Cambridge, MA: Academic Press.

[eva12961-bib-0042] Dumont, B. L. (2015). Interlocus gene conversion explains at least 2.7% of single nucleotide variants in human segmental duplications. BMC Genomics, 16(1), 456 10.1186/s12864-015-1681-3 26077037 PMC4467073

[eva12961-bib-0043] Durand, É. , Gagnon‐Arsenault, I. , Hallin, J. , Hatin, I. , Dubé, A. K. , Nielly‐Thibault, L. , … Landry, C. R. (2019). Turnover of ribosome‐associated transcripts from de novo ORFs produces gene‐like characteristics available for de novo gene emergence in wild yeast populations. Genome Research, 29(6), 932–943. 10.1101/gr.239822.118 31152050 PMC6581059

[eva12961-bib-0044] Eberlein, C. , Hénault, M. , Fijarczyk, A. , Charron, G. , Bouvier, M. , Kohn, L. M. , … Landry, C. R. (2019). Hybridization is a recurrent evolutionary stimulus in wild yeast speciation. Nature Communications, 10(1), 923 10.1038/s41467-019-08809-7 PMC6389940 30804385

[eva12961-bib-0045] Ehrenberg, C. (1835). Dritter Beitrag zur Erkenntniss grosser Organisation in der Richtung des kleinsten Raumes. Physikalische Abhandlungen Der Koeniglichen Akademie Der Wissenschaften Zu Berlin Aus Den Jahren, 1833–1835, 143–336.

[eva12961-bib-0046] Elde, N. C. , Child, S. J. , Geballe, A. P. , & Malik, H. S. (2009). Protein kinase R reveals an evolutionary model for defeating viral mimicry. Nature, 457(7228), 485–489. 10.1038/nature07529 19043403 PMC2629804

[eva12961-bib-0047] Emery, C. (1890). Alcune considerazioni sulla fauna mirmecologica dell’Africa. Bollettino della Societa Entomologica Italiana, 21, 69–75.

[eva12961-bib-0048] Enard, D. , Cai, L. , Gwennap, C. , & Petrov, D. A. (2016). Viruses are a dominant driver of protein adaptation in mammals. eLife, 5, 27187613 10.7554/eLife.12469 PMC4869911 27187613

[eva12961-bib-0049] Erwig, L. P. , & Gow, N. A. R. (2016). Interactions of fungal pathogens with phagocytes. Nature Reviews. Microbiology, 14(3), 163–176. 10.1038/nrmicro.2015.21 26853116

[eva12961-bib-0050] Flemming, H.‐C. , Wingender, J. , Szewzyk, U. , Steinberg, P. , Rice, S. A. , & Kjelleberg, S. (2016). Biofilms: An emergent form of bacterial life. Nature Reviews Microbiology, 14(9), 563–575. 10.1038/nrmicro.2016.94 27510863

[eva12961-bib-0051] Force, A. , Lynch, M. , Pickett, F. B. , Amores, A. , Yan, Y. L. , & Postlethwait, J. (1999). Preservation of duplicate genes by complementary, degenerative mutations. Genetics, 151(4), 1531–1545. 10101175 10.1093/genetics/151.4.1531 PMC1460548

[eva12961-bib-0052] Forel, A. (1898). La parabiose chez les fourmis. Bulletin De La Société Vaudoise Des Sciences Naturelles, 34, 380–384.

[eva12961-bib-0053] Fortuna, M. A. , Stouffer, D. B. , Olesen, J. M. , Jordano, P. , Mouillot, D. , Krasnov, B. R. , … Bascompte, J. (2010). Nestedness versus modularity in ecological networks: Two sides of the same coin? The Journal of Animal Ecology, 79(4), 811–817. 10.1111/j.1365-2656.2010.01688.x 20374411

[eva12961-bib-0054] Francino, M. P. (2005). An adaptive radiation model for the origin of new gene functions. Nature Genetics, 37, 573–578. 10.1038/ng1579 15920518

[eva12961-bib-0055] Furness, A. I. , Morrison, K. R. , Orr, T. J. , Arendt, J. D. , & Reznick, D. N. (2015). Reproductive mode and the shifting arenas of evolutionary conflict. Annals of the New York Academy of Sciences, 1360, 75–100. 10.1111/nyas.12835 26284738

[eva12961-bib-0056] Gagnon‐Arsenault, I. , Marois Blanchet, F.‐C. , Rochette, S. , Diss, G. , Dubé, A. K. , & Landry, C. R. (2013). Transcriptional divergence plays a role in the rewiring of protein interaction networks after gene duplication. Journal of Proteomics, 81, 112–125. 10.1016/j.jprot.2012.09.038 23063722

[eva12961-bib-0057] Guan, Y. , Dunham, M. J. , & Troyanskaya, O. G. (2007). Functional analysis of gene duplications in *Saccharomyces cerevisiae* . Genetics, 175(2), 933–943. 10.1534/genetics.106.064329 17151249 PMC1800624

[eva12961-bib-0058] Günther, A. (1864). Catalogue of the fishes in the British Museum. Catalogue of the Physostomi, containing the families Siluridae, Characinidae, Haplochitonidae, Sternoptychidae, Scopelidae, Stomiatidae in the collection of the British Museum. Catalogue of the Fishes in the British Museum, 5, i – xxii.

[eva12961-bib-0059] Günther, A. (1867). On the fishes of the states of Central America, founded upon specimens collected in fresh and marine waters of various parts of that country by Messrs. Salvin and Godman and Capt. J. M. Dow. Proceedings of the Zoological Society of London, 600–604.

[eva12961-bib-0060] Gutiérrez‐Pinto, N. , Cuervo, A. M. , Miranda, J. , Pérez‐Emán, J. L. , Brumfield, R. T. , & Cadena, C. D. (2012). Non‐monophyly and deep genetic differentiation across low‐elevation barriers in a neotropical montane bird (*Basileuterus tristriatus*; Aves: Parulidae). Molecular Phylogenetics and Evolution, 64(1), 156–165. 10.1016/j.ympev.2012.03.011 22484358

[eva12961-bib-0061] Hakes, L. , Pinney, J. W. , Lovell, S. C. , Oliver, S. G. , & Robertson, D. L. (2007). All duplicates are not equal: The difference between small‐scale and genome duplication. Genome Biology, 8(10), R209 10.1186/gb-2007-8-10-r209 17916239 PMC2246283

[eva12961-bib-0062] Hanifin, C. T. , Brodie, E. D. , & Brodie, E. D. (2008). Phenotypic mismatches reveal escape from arms‐race coevolution. PLoS Biology, 6(3), e60 10.1371/journal.pbio.0060060 18336073 PMC2265764

[eva12961-bib-0063] Harpak, A. , Lan, X. , Gao, Z. , & Pritchard, J. K. (2017). Frequent nonallelic gene conversion on the human lineage and its effect on the divergence of gene duplicates. Proceedings of the National Academy of Sciences of the United States of America, 114(48), 12779–12784. 10.1073/pnas.1708151114 29138319 PMC5715747

[eva12961-bib-0064] Harvey, E. , Gounand, I. , Ward, C. L. , & Altermatt, F. (2017). Bridging ecology and conservation: From ecological networks to ecosystem function. Journal of Applied Ecology, 54(2), 371–379. 10.1111/1365-2664.12769

[eva12961-bib-0065] Havilio, M. , Levanon, E. Y. , Lerman, G. , Kupiec, M. , & Eisenberg, E. (2005). Evidence for abundant transcription of non‐coding regions in the *Saccharomyces cerevisiae* genome. BMC Genomics, 6, 93. 15960846 10.1186/1471-2164-6-93 PMC1181813

[eva12961-bib-0066] He, X. , & Zhang, J. (2005). Rapid subfunctionalization accompanied by prolonged and substantial neofunctionalization in duplicate gene evolution. Genetics, 169(2), 1157–1164. 10.1534/genetics.104.037051 15654095 PMC1449125

[eva12961-bib-0067] He, X. , & Zhang, J. (2006). Why do hubs tend to be essential in protein networks? PLoS Genetics, 2(6), e88 10.1371/journal.pgen.0020088 16751849 PMC1473040

[eva12961-bib-0068] Hébert, F. O. , Grambauer, S. , Barber, I. , Landry, C. R. , & Aubin‐Horth, N. (2017). Major host transitions are modulated through transcriptome‐wide reprogramming events in *Schistocephalus solidus*, a threespine stickleback parasite. Molecular Ecology, 26(4), 1118–1130. 10.1111/mec.13970 27997044

[eva12961-bib-0069] Henikoff, S. , Ahmad, K. , & Malik, H. S. (2001). The centromere paradox: Stable inheritance with rapidly evolving DNA. Science, 293(5532), 1098–1102. 10.1126/science.1062939 11498581

[eva12961-bib-0070] Hewitt, G. M. (1976). Meiotic drive for B‐chromosomes in the primary oocytes of *Myrmeleotettix maculatus* (Orthopera: Acrididae). Chromosoma, 56(4), 381–391. 949923 10.1007/BF00292957

[eva12961-bib-0071] Hsiao, T.‐L. , & Vitkup, D. (2008). Role of duplicate genes in robustness against deleterious human mutations. PLoS Genetics, 4(3), e1000014 10.1371/journal.pgen.1000014 18369440 PMC2265532

[eva12961-bib-0072] Hubbell, S. P. (2006). Neutral theory and the evolution of ecological equivalence. Ecology, 87(6), 1387–1398. 10.1890/0012-9658(2006)87[1387:NTATEO]2.0.CO;2 16869413

[eva12961-bib-0073] Ispolatov, I. , Yuryev, A. , Mazo, I. , & Maslov, S. (2005). Binding properties and evolution of homodimers in protein–protein interaction networks. Nucleic Acids Research, 33(11), 3629–3635. 10.1093/nar/gki678 15983135 PMC1160523

[eva12961-bib-0074] Jacob, F. (1993). The logic of life: A history of heredity. Princeton: Princeton University Press.

[eva12961-bib-0075] Jacob, F. , & Monod, J. (1961). Genetic regulatory mechanisms in the synthesis of proteins. Journal of Molecular Biology, 3, 318–356. 10.1016/S0022-2836(61)80072-7 13718526

[eva12961-bib-0076] Jacobs, F. M. J. , Greenberg, D. , Nguyen, N. , Haeussler, M. , Ewing, A. D. , Katzman, S. , … Haussler, D. (2014). An evolutionary arms race between KRAB zinc‐finger genes ZNF91/93 and SVA/L1 retrotransposons. Nature, 516(7530), 242–245. 25274305 10.1038/nature13760 PMC4268317

[eva12961-bib-0077] Jarau, S. , & Hrncir, M. (2009). Food exploitation by social insects: Ecological, behavioral, and theoretical approaches. Boca Raton, FL: CRC Press.

[eva12961-bib-0078] Ji, Z. , Song, R. , Regev, A. , & Struhl, K. (2015). Many lncRNAs, 5’UTRs, and pseudogenes are translated and some are likely to express functional proteins. eLife, 4, e08890 10.7554/eLife.08890 26687005 PMC4739776

[eva12961-bib-0079] Johnson, C. W. (2006). What are emergent properties and how do they affect the engineering of complex systems? Reliability Engineering & System Safety, 91(12), 1475–1481. 10.1016/j.ress.2006.01.008

[eva12961-bib-0080] Johnson, F. H. , & Shunk, I. V. (1936). An Interesting New Species of Luminous Bacteria. Journal of Bacteriology, 31(6), 585–593. 16559916 10.1128/jb.31.6.585-593.1936 PMC543750

[eva12961-bib-0081] Kalamara, M. , Spacapan, M. , Mandic‐Mulec, I. , & Stanley‐Wall, N. R. (2018). Social behaviours by Bacillus subtilis: Quorum sensing, kin discrimination and beyond. Molecular Microbiology, 110(6), 863–878. 30218468 10.1111/mmi.14127 PMC6334282

[eva12961-bib-0082] Kaplan, M. (2011). Ancient bats got in a flap over food. Nature, 10.1038/nature.2011.9304

[eva12961-bib-0083] Kapranov, P. , Cawley, S. E. , Drenkow, J. , Bekiranov, S. , Strausberg, R. L. , Fodor, S. P. A. , & Gingeras, T. R. (2002). Large‐scale transcriptional activity in chromosomes 21 and 22. Science, 296(5569), 916–919. 11988577 10.1126/science.1068597

[eva12961-bib-0084] Katz, Y. , Tunstrøm, K. , Ioannou, C. C. , Huepe, C. , & Couzin, I. D. (2011). Inferring the structure and dynamics of interactions in schooling fish. Proceedings of the National Academy of Sciences of the United States of America, 108(46), 18720–18725. 10.1073/pnas.1107583108 21795604 PMC3219116

[eva12961-bib-0085] Keller, E. F. (2002). The century of the gene. Cambridge, MA: Harvard University Press.

[eva12961-bib-0086] Kocher, T. D. (2004). Adaptive evolution and explosive speciation: The cichlid fish model. Nature Reviews Genetics, 5(4), 288–298. 10.1038/nrg1316 15131652

[eva12961-bib-0087] Kranzusch, P. J. , Wilson, S. C. , Lee, A. S. Y. , Berger, J. M. , Doudna, J. A. , & Vance, R. E. (2015). Ancient origin of cGAS‐STING reveals mechanism of universal 2′,3′ cGAMP signaling. Molecular Cell, 59(6), 891–903. 10.1016/j.molcel.2015.07.022 26300263 PMC4575873

[eva12961-bib-0088] Kursel, L. E. , & Malik, H. S. (2018). The cellular mechanisms and consequences of centromere drive. Current Opinion in Cell Biology, 52, 58–65. 10.1016/j.ceb.2018.01.011 29454259 PMC5988936

[eva12961-bib-0089] Lamb, T. D. , Collin, S. P. , & Pugh, E. N. Jr (2007). Evolution of the vertebrate eye: Opsins, photoreceptors, retina and eye cup. Nature Reviews Neuroscience, 8(12), 960–976. 10.1038/nrn2283 18026166 PMC3143066

[eva12961-bib-0090] Lambert, M. J. , Cochran, W. O. , Wilde, B. M. , Olsen, K. G. , & Cooper, C. D. (2015). Evidence for widespread subfunctionalization of splice forms in vertebrate genomes. Genome Research, 25(5), 624–632. 10.1101/gr.184473.114 25792610 PMC4417111

[eva12961-bib-0091] Landi, P. , Minoarivelo, H. O. , Brännström, Å. , Hui, C. , & Dieckmann, U. (2018). Complexity and stability of ecological networks: A review of the theory. Population Ecology, 60(4), 319–345. 10.1007/s10144-018-0628-3

[eva12961-bib-0092] Landry, C. R. , Levy, E. D. , Abd Rabbo, D. , Tarassov, K. , & Michnick, S. W. (2013). Extracting insight from noisy cellular networks. Cell, 155(5), 983–989. 10.1016/j.cell.2013.11.003 24267884

[eva12961-bib-0093] Larracuente, A. M. , & Presgraves, D. C. (2012). The selfish segregation distorter gene complex of *Drosophila melanogaster* . Genetics, 192(1), 33–53. 22964836 10.1534/genetics.112.141390 PMC3430544

[eva12961-bib-0094] Leducq, J.‐B. , Nielly‐Thibault, L. , Charron, G. , Eberlein, C. , Verta, J.‐P. , Samani, P. , … Landry, C. R. (2016). Speciation driven by hybridization and chromosomal plasticity in a wild yeast. Nature Microbiology, 1, 15003 10.1038/nmicrobiol.2015.3 27571751

[eva12961-bib-0095] Lehtonen, T. K. (2008). Convict cichlids benefit from close proximity to another species of cichlid fish. Biology Letters, 4(6), 610–612. 10.1098/rsbl.2008.0378 18762472 PMC2614157

[eva12961-bib-0096] Lesson, R. P. (1827). Manuel de mammalogie, ou histoire naturelle des mammiferes (Vol. 1). Paris, France: Roret libraire.

[eva12961-bib-0097] Levine, M. T. , Jones, C. D. , Kern, A. D. , Lindfors, H. A. , & Begun, D. J. (2006). Novel genes derived from noncoding DNA in *Drosophila melanogaster* are frequently X‐linked and exhibit testis‐biased expression. Proceedings of the National Academy of Sciences of the United States of America, 103(26), 9935–9939. 10.1073/pnas.0509809103 16777968 PMC1502557

[eva12961-bib-0098] Lien, S. , Koop, B. F. , Sandve, S. R. , Miller, J. R. , Kent, M. P. , Nome, T. , … Davidson, W. S. (2016). The Atlantic salmon genome provides insights into rediploidization. Nature, 533(7602), 200–205. 27088604 10.1038/nature17164 PMC8127823

[eva12961-bib-0099] Lincoln, R. J. (1998). A dictionary of ecology, evolution and systematics. Cambridge: Cambridge University Press.

[eva12961-bib-0100] Linnaeus, C. (1758). Systema naturae per regna tria naturae, secundum classes, ordines, genera, species, cum characteribus, differentiis, synonymis, locis (Vol. 1). Holmiae. (Salvius).

[eva12961-bib-0101] Liu, Y. , Xie, D. , Han, L. , Bai, H. , Li, F. , Wang, S. , & Bo, X. (2015). EHFPI: A database and analysis resource of essential host factors for pathogenic infection. Nucleic Acids Research, 43(D1), D946–D955. 10.1093/nar/gku1086 25414353 PMC4383917

[eva12961-bib-0102] Longino, J. T. (2003). The Crematogaster (Hymenoptera, Formicidae, Myrmicinae) of Costa Rica. Zootaxa, 151(1), 1–150.

[eva12961-bib-0103] Lukatsky, D. B. , Shakhnovich, B. E. , Mintseris, J. , & Shakhnovich, E. I. (2007). Structural similarity enhances interaction propensity of proteins. Journal of Molecular Biology, 365(5), 1596–1606. 10.1016/j.jmb.2006.11.020 17141268 PMC2735088

[eva12961-bib-0104] Lukatsky, D. B. , Zeldovich, K. B. , & Shakhnovich, E. I. (2006). Statistically enhanced self‐attraction of random patterns. Physical Review Letters, 97(17), 178101 10.1103/PhysRevLett.97.178101 17155509

[eva12961-bib-0105] Lynch, M. , & Conery, J. S. (2000). The evolutionary fate and consequences of duplicate genes. Science, 290(5494), 1151–1155. 11073452 10.1126/science.290.5494.1151

[eva12961-bib-0106] Lynch, M. , & Force, A. (2000). The probability of duplicate gene preservation by subfunctionalization. Genetics, 154(1), 459–473.10629003

[eva12961-bib-0107] Marceau, C. D. , Puschnik, A. S. , Majzoub, K. , Ooi, Y. S. , Brewer, S. M. , Fuchs, G. , … Carette, J. E. (2016). Genetic dissection of flaviviridae host factors through genome‐scale CRISPR screens. Nature, 535(7610), 159–163. 27383987 10.1038/nature18631 PMC4964798

[eva12961-bib-0108] Marchant, A. , Cisneros, A. F. , Dubé, A. K. , Gagnon‐Arsenault, I. , Ascencio, D. , Jain, H. , … Landry, C. R. (2019). The role of structural pleiotropy and regulatory evolution in the retention of heteromers of paralogs. Elife, 8, e46754. 31454312 10.7554/eLife.46754 PMC6711710

[eva12961-bib-0109] Marianayagam, N. J. , Sunde, M. , & Matthews, J. M. (2004). The power of two: Protein dimerization in biology. Trends in Biochemical Sciences, 29(11), 618–625. 10.1016/j.tibs.2004.09.006 15501681

[eva12961-bib-0110] Mavárez, J. , Salazar, C. A. , Bermingham, E. , Salcedo, C. , Jiggins, C. D. , & Linares, M. (2006). Speciation by hybridization in Heliconius butterflies. Nature, 441(7095), 868–871. 16778888 10.1038/nature04738

[eva12961-bib-0111] Mayr, E. (1942). Systematics and the origin of species. New York, NY: Columbia University Press.

[eva12961-bib-0112] Mayr, E. (1947). Ecological factors in speciation. Evolution; International Journal of Organic Evolution, 1(4), 263–288. 10.1111/j.1558-5646.1947.tb02723.x

[eva12961-bib-0113] McClintock, B. (1950). The origin and behavior of mutable loci in maize. Proceedings of the National Academy of Sciences of the United States of America, 36(6), 344–355. 10.1073/pnas.36.6.344 15430309 PMC1063197

[eva12961-bib-0114] McDougald, D. , Srinivasan, S. , Rice, S. A. , & Kjelleberg, S. (2003). Signal‐mediated cross‐talk regulates stress adaptation in Vibrio species. Microbiology, 149(Pt 7), 1923–1933. 10.1099/mic.0.26321-0 12855743

[eva12961-bib-0115] McLaughlin, R. N. Jr , & Malik, H. S. (2017). Genetic conflicts: The usual suspects and beyond. The Journal of Experimental Biology, 220(Pt 1), 6–17. 10.1242/jeb.148148 28057823 PMC5278622

[eva12961-bib-0116] Meier, J. I. , Marques, D. A. , Mwaiko, S. , Wagner, C. E. , Excoffier, L. , & Seehausen, O. (2017). Ancient hybridization fuels rapid cichlid fish adaptive radiations. Nature Communications, 8(1), 14363 10.1038/ncomms14363 PMC5309898 28186104

[eva12961-bib-0117] Menzel, F. , Blüthgen, N. , Tolasch, T. , Conrad, J. , Beifuß, U. , Beuerle, T. , & Schmitt, T. (2013). Crematoenones – A novel substance class exhibited by ants functions as appeasement signal. Frontiers in Zoology, 10(1), 32– 10.1186/1742-9994-10-32 23742696 PMC3691653

[eva12961-bib-0118] Menzel, F. , Linsenmair, K. E. , & Blüthgen, N. (2008). Selective interspecific tolerance in tropical Crematogaster‐Camponotus associations. Animal Behaviour, 75(3), 837–846. 10.1016/j.anbehav.2007.07.005

[eva12961-bib-0119] Migula, W. (1900). System der bakterien (Vol. 2). Jena, Germany: Fischer.

[eva12961-bib-0120] Miller, M. B. , & Bassler, B. L. (2001). Quorum sensing in bacteria. Annual Review of Microbiology, 55, 165–199. 10.1146/annurev.micro.55.1.165 11544353

[eva12961-bib-0121] Miura, K. (2018). An overview of current methods to confirm protein‐protein interactions. Protein and Peptide Letters, 25(8), 728 10.2174/0929866525666180821122240 30129399 PMC6204658

[eva12961-bib-0122] Mock, D. W. (1984). Siblicidal aggression and resource monopolization in birds. Science, 225(4663), 731–733. 10.1126/science.225.4663.731 17810292

[eva12961-bib-0123] Muller, H. (1942). Isolating mechanisms, evolution, and temperature. Biological Symposium, 6, 71–125.

[eva12961-bib-0124] Nathan, A. , & Barbosa, V. C. (2008). V‐like formations in flocks of artificial birds. Artificial Life, 14(2), 179–188. 10.1162/artl.2008.14.2.179 18331189

[eva12961-bib-0125] Nielly‐Thibault, L. , & Landry, C. R. (2019). Differences between the raw material and the products of de novo gene birth can result from mutational biases. Genetics, 212(4), 1353–1366. 31227545 10.1534/genetics.119.302187 PMC6707459

[eva12961-bib-0126] Olson, M. E. , Arroyo‐Santos, A. , & Vergara‐Silva, F. (2019). A user’s guide to metaphors in ecology and evolution. Trends in Ecology & Evolution, 34(7), 605–615. 10.1016/j.tree.2019.03.001 31000371

[eva12961-bib-0127] Otto, S. P. , & Whitton, J. (2000). Polyploid incidence and evolution. Annual Review of Genetics, 34, 401–437. 10.1146/annurev.genet.34.1.401 11092833

[eva12961-bib-0128] Pedrini, N. , Ortiz‐Urquiza, A. , Huarte‐Bonnet, C. , Fan, Y. , Juárez, M. P. , & Keyhani, N. O. (2015). Tenebrionid secretions and a fungal benzoquinone oxidoreductase form competing components of an arms race between a host and pathogen. Proceedings of the National Academy of Sciences of the United States of America, 112(28), E3651–E3660. 10.1073/pnas.1504552112 26056261 PMC4507192

[eva12961-bib-0129] Pocock, M. J. O. , Evans, D. M. , & Memmott, J. (2012). The robustness and restoration of a network of ecological networks. Science, 335(6071), 973–977. 10.1126/science.1214915 22363009

[eva12961-bib-0130] Queller, D. C. (2014). Joint phenotypes, evolutionary conflict and the fundamental theorem of natural selection. Philosophical Transactions of the Royal Society of London. Series B, Biological Sciences, 369(1642), 20130423 10.1098/rstb.2013.0423 24686940 PMC3982670

[eva12961-bib-0131] Queller, D. C. , & Strassmann, J. E. (2018). Evolutionary conflict. Annual Review of Ecology, Evolution, and Systematics, 49(1), 73–93. 10.1146/annurev-ecolsys-110617-062527

[eva12961-bib-0132] Reams, A. B. , & Roth, J. R. (2015). Mechanisms of gene duplication and amplification. Cold Spring Harbor Perspectives in Biology, 7(2), a016592 10.1101/cshperspect.a016592 25646380 PMC4315931

[eva12961-bib-0133] Redondo, T. , Romero, J. M. , Díaz‐Delgado, R. , & Nagy, J. (2019). Broodmate aggression and life history variation in accipitrid birds of prey. Ecology and Evolution, 9(16), 9185–9206. 10.1002/ece3.5466 31463015 PMC6706193

[eva12961-bib-0134] Romanuk, T. N. , Zhou, Y. , Brose, U. , Berlow, E. L. , Williams, R. J. , & Martinez, N. D. (2009). Predicting invasion success in complex ecological networks. Philosophical Transactions of the Royal Society of London. Series B, Biological Sciences, 364(1524), 1743–1754. 10.1098/rstb.2008.0286 19451125 PMC2685429

[eva12961-bib-0135] Rubin, G. M. , Kidwell, M. G. , & Bingham, P. M. (1982). The molecular basis of P‐M hybrid dysgenesis: The nature of induced mutations. Cell, 29(3), 987–994. 10.1016/0092-8674(82)90462-7 6295640

[eva12961-bib-0136] Ruiz‐Orera, J. , Verdaguer‐Grau, P. , Villanueva‐Cañas, J. L. , Messeguer, X. , & Mar Albà, M. (2018). Translation of neutrally evolving peptides provides a basis for de novo gene evolution. Nature Ecology & Evolution, 2(5), 890–896. 10.1038/s41559-018-0506-6 29556078

[eva12961-bib-0137] Saeed, R. , & Deane, C. M. (2006). Protein protein interactions, evolutionary rate, abundance and age. BMC Bioinformatics, 7, 128. 16533385 10.1186/1471-2105-7-128 PMC1431566

[eva12961-bib-0138] Sandve, S. R. , Rohlfs, R. V. , & Hvidsten, T. R. (2018). Subfunctionalization versus neofunctionalization after whole‐genome duplication. Nature Genetics, 50(7), 908–909. 10.1038/s41588-018-0162-4 29955176

[eva12961-bib-0139] Sawyer, S. L. , Emerman, M. , & Malik, H. S. (2007). Discordant evolution of the adjacent antiretroviral genes TRIM22 and TRIM5 in mammals. PLoS Path, 3(12), e197 10.1371/journal.ppat.0030197 PMC2151084 18159944

[eva12961-bib-0140] Schroeter, J. (1872). Ueber einige durch Bacterien gebildete Pigmente. Beiträge Zur Biologie Der Pflanzen, 1(2), 109–126.

[eva12961-bib-0141] Smith, J. M. (1970). Natural selection and the concept of a protein space. Nature, 225(5232), 563–564. 5411867 10.1038/225563a0

[eva12961-bib-0142] Smith, R. S. , & Iglewski, B. H. (2003). P. aeruginosa quorum‐sensing systems and virulence. Current Opinion in Microbiology, 6(1), 56–60. 10.1016/S1369-5274(03)00008-0 12615220

[eva12961-bib-0143] Soltis, P. S. , & Soltis, D. E. (2009). The role of hybridization in plant speciation. Annual Review of Plant Biology, 60, 561–588. 10.1146/annurev.arplant.043008.092039 19575590

[eva12961-bib-0144] Sterner, R. W. , & Elser, J. J. (2002). Ecological stoichiometry: The biology of elements from molecules to the biosphere. Princeton: Princeton University Press.

[eva12961-bib-0145] Stoddard, M. C. , & Stevens, M. (2011). Avian vision and the evolution of egg color mimicry in the common cuckoo. Evolution; International Journal of Organic Evolution, 65(7), 2004–2013. 10.1111/j.1558-5646.2011.01262.x 21729055

[eva12961-bib-0146] Strauss, S. Y. , Lau, J. A. , & Carroll, S. P. (2006). Evolutionary responses of natives to introduced species: What do introductions tell us about natural communities? Ecology Letters, 9(3), 357–374. 10.1111/j.1461-0248.2005.00874.x 16958902

[eva12961-bib-0147] Tarassov, K. , Messier, V. , Landry, C. R. , Radinovic, S. , Molina, M. M. S. , Shames, I. , … Michnick, S. W. (2008). An in vivo map of the yeast protein interactome. Science, 320(5882), 1465–1470. 10.1126/science.1153878 18467557

[eva12961-bib-0148] Tautz, D. (2014). The discovery of de novo gene evolution. Perspectives in Biology and Medicine, 57(1), 149–161. 10.1353/pbm.2014.0006 25345708

[eva12961-bib-0149] Teeling, E. C. , Springer, M. S. , Madsen, O. , Bates, P. , O'brien, S. J. , & Murphy, W. J. (2005). A molecular phylogeny for bats illuminates biogeography and the fossil record. Science, 307(5709), 580–584. 15681385 10.1126/science.1105113

[eva12961-bib-0150] Ter Hofstede, H. M. , & Ratcliffe, J. M. (2016). Evolutionary escalation: The bat‐moth arms race. The Journal of Experimental Biology, 219(Pt 11), 1589–1602. 10.1242/jeb.086686 27252453

[eva12961-bib-0151] Thewissen, J. G. M. , Cooper, L. N. , George, J. C. , & Bajpai, S. (2009). From land to water: The origin of whales, dolphins, and porpoises. Evolution: Education and Outreach, 2(2), 272.

[eva12961-bib-0152] Thompson, K. A. , Rieseberg, L. H. , & Schluter, D. (2018). Speciation and the city. Trends in Ecology & Evolution, 33(11), 815–826. 10.1016/j.tree.2018.08.007 30297245

[eva12961-bib-0153] Toll‐Riera, M. , Bosch, N. , Bellora, N. , Castelo, R. , Armengol, L. , Estivill, X. , & Albà, M. M. (2009). Origin of primate orphan genes: A comparative genomics approach. Molecular Biology and Evolution, 26(3), 603–612. 10.1093/molbev/msn281 19064677

[eva12961-bib-0154] Turner, B. C. , & Perkins, D. D. (1991). Meiotic drive in neurospora and other fungi. The American Naturalist, 137(3), 416–429. 10.1086/285174

[eva12961-bib-0155] van Gestel, J. , Weissing, F. J. , Kuipers, O. P. , & Kovács, A. T. (2014). Density of founder cells affects spatial pattern formation and cooperation in *Bacillus subtilis* biofilms. The ISME Journal, 8(10), 2069–2079. 10.1038/ismej.2014.52 24694715 PMC4184017

[eva12961-bib-0156] van Meer, G. , Voelker, D. R. , & Feigenson, G. W. (2008). Membrane lipids: Where they are and how they behave. Nature Reviews. Molecular Cell Biology, 9(2), 112–124. 10.1038/nrm2330 18216768 PMC2642958

[eva12961-bib-0157] Van Valen, L. (1973). A new evolutionary law. Evolutionary Theory, 1, 1–30.

[eva12961-bib-0158] Vuillemin, P. (1901). Les blastomycetes pathogenes. Revue Generale Des Sciences Pures et Appliquees et Bulletin de l’Association Francaise Pour L'avancement Des Sciences, 12, 732–751.

[eva12961-bib-0159] Werren, J. H. (2011). Selfish genetic elements, genetic conflict, and evolutionary innovation. Proceedings of the National Academy of Sciences of the United States of America, 108(Suppl 2), 10863–10870. 10.1073/pnas.1102343108 21690392 PMC3131821

[eva12961-bib-0160] Wicker, T. , Sabot, F. , Hua‐Van, A. , Bennetzen, J. L. , Capy, P. , Chalhoub, B. , … Schulman, A. H. (2007). A unified classification system for eukaryotic transposable elements. Nature Reviews Genetics, 8(12), 973–982. 10.1038/nrg2165 17984973

[eva12961-bib-0161] Willis, A. J. (1997). The ecosystem: An evolving concept viewed historically. Functional Ecology, 11(2), 268–271.

[eva12961-bib-0162] Wilson, B. A. , & Masel, J. (2011). Putatively noncoding transcripts show extensive association with ribosomes. Genome Biology and Evolution, 3, 1245–1252. 10.1093/gbe/evr099 21948395 PMC3209793

[eva12961-bib-0163] Wuchty, S. (2014). Controllability in protein interaction networks. Proceedings of the National Academy of Sciences of the United States of America, 111(19), 7156–7160. 10.1073/pnas.1311231111 24778220 PMC4024882

[eva12961-bib-0164] Wyman, R. L. , & Ward, J. A. (1972). A cleaning symbiosis between the cichlid fishes *Etroplus maculatus* and *Etroplus suratensis*. I. Description and possible evolution. Copeia, 1972(4), 834–838.

[eva12961-bib-0165] Yu, D. , Kim, M. , Xiao, G. , & Hwang, T. H. (2013). Review of biological network data and its applications. Genomics & Informatics, 11(4), 200–210. 10.5808/GI.2013.11.4.200 24465231 PMC3897847

[eva12961-bib-0166] Zaragoza, O. , & Nielsen, K. (2013). Titan cells in Cryptococcus neoformans: Cells with a giant impact. Current Opinion in Microbiology, 16(4), 409–413. 10.1016/j.mib.2013.03.006 23588027 PMC3723695

[eva12961-bib-0167] Zhang, J. (2003). Evolution by gene duplication: An update. Trends in Ecology & Evolution, 18(6), 292–298. 10.1016/S0169-5347(03)00033-8

[eva12961-bib-0168] Zhang, L. I. , Ren, Y. , Yang, T. , Li, G. , Chen, J. , Gschwend, A. R. , … Long, M. (2019). Rapid evolution of protein diversity by de novo origination in Oryza. Nature Ecology & Evolution, 3(4), 679–690. 10.1038/s41559-019-0822-5 30858588

